# Recombinant Antibody-Based and Computer-Aided Comprehensive Analysis of Antibody’s Equivalent Recognition Mechanism of Alternariol and Alternariol Monomethyl Ether

**DOI:** 10.3389/fchem.2022.871659

**Published:** 2022-04-14

**Authors:** Zile Wang, Ling Chen, Pimiao Zheng, Jianyi Wang, Zhenhui Ren, Huixia Zhang, Liang Zhang, Haiyang Jiang

**Affiliations:** ^1^ Department of Veterinary Pharmacology and Toxicology, Beijing Key Laboratory of Detection Technology for Animal-Derived Food Safety, Beijing Laboratory for Food Quality and Safety, College of Veterinary Medicine, China Agricultural University, Beijing, China; ^2^ China Institution of Veterinary Drug Control, Beijing, China; ^3^ College of Veterinary Medicine, Shandong Agricultural University, Taian, China

**Keywords:** molecular interaction, recognition mechanism, alternariol, alternariol monomethyl ether, single-chain antibody fragment

## Abstract

Alternariol (AOH) and alternariol monomethyl ether (AME) are two main *Alternaria* mycotoxins that endanger human health. In this study, a single-chain antibody fragment (scFv) capable of equivalently and specifically recognizing AOH and AME was first expressed, and its equivalent recognition mechanism was discussed. According to molecular docking and dynamic simulation, the C9 site, which was always exposed outside the binding cavity, made the structural differences between AOH and AME negligible. Due to the high similarity of structures, AOH and AME interacted with almost the same amino acids on the scFv; thus, the same interaction mode and interaction force were produced. This was considered to be the most critical reason for the equivalent recognition. Thus, the exposure of common structures was considered a potential strategy to obtain the equivalent recognition antibodies, and C9 was considered the key site in the process of hapten modification. These results laid a theoretical foundation for further research on antibodies against *Alternaria* mycotoxins. It could promote the rapid detection of AOH and AME in food and provide a new idea for targeted preparation of antibodies that could recognize multiple hazards with similar structures.

## Graphical Abstract

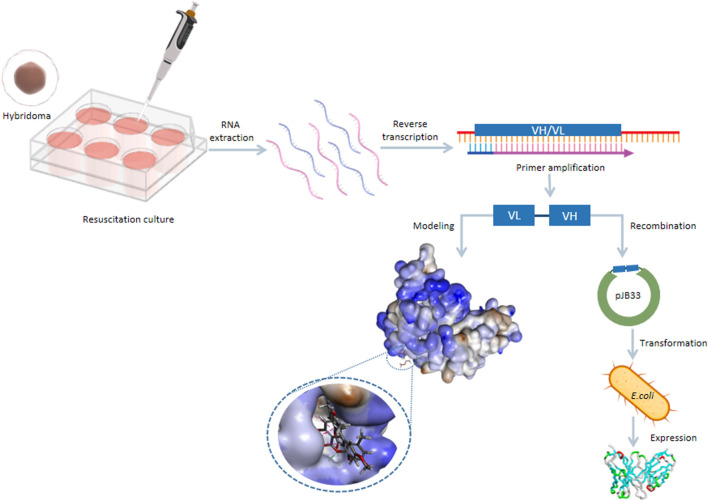

## Introduction

**FIGURE 1:**
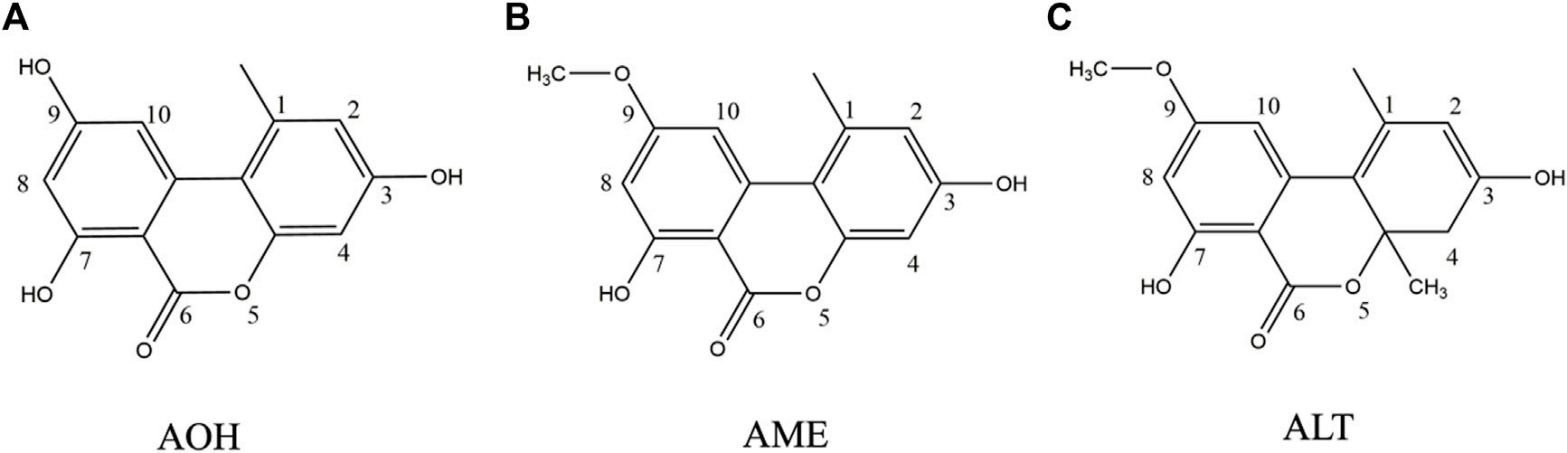
Structure of three main *Alternaria alternata* mycotoxins. **(A)** Alternariol. **(B)** Alternariol monomethyl ether. **(C)** Altenuene.

Mycotoxins have teratogenic, mutagenic, and carcinogenic effects even at low concentrations, and long-term consumption of food contaminated with mycotoxins may cause serious consequences, such as cancer (Bhat et al., 2010). In recent years, “emerging mycotoxins” including *Alternaria* mycotoxins are attracting increasing public attention due to their potential toxicity (Gruber-Dorninger et al., 2017). *Alternaria* mycotoxins are secondary metabolites produced by *Alternaria* species. According to the European Food Safety Authority (EFSA), alternariol (AOH) and alternariol monomethyl ether (AME) are two main *Alternaria* mycotoxins that endanger human health (ESFA, 2011). They belong to dibenzo-α-pyrone derivatives and structurally resemble altenuene (ALT) (Figure 1). *Alternaria* mycotoxins are widely distributed in nature, and they could be detected in milk, cereals, fruits, vegetables, spices, and herbs (Gambacorta et al., 2019; Li et al., 2020; Orina et al., 2021; Akinyemi et al., 2022). It is reported that *Alternaria* mycotoxins are thermally stable (Estiarte et al., 2018). AOH and AME showed remarkable cytotoxicity to mammalian cells *in vitro* (Solhaug et al., 2014), and they had a synergistic effect when used together in high concentrations. Tenuazonic acid (TeA) exhibits toxicity against several animal species such as mice, chickens, and dogs (EFSA, 2011). In addition, the incidence of esophageal cancer is higher in areas with high AOH and AME food contamination. Currently, there is no regulation for *Alternaria* mycotoxins worldwide except for the Bavarian Health and Food Safety Authority which established a limit of 500 μg/kg for TeA in sorghum/millet-based infant food (Gambacorta et al., 2019). However, the defined threshold of toxicological concern (TTC) of AOH and AME was 2.5 ng/kg body weight, while TeA was 1,500 ng/kg body weight (ESFA, 2011). Therefore, it is of great significance to strengthen the monitoring of the incidence of *Alternaria* mycotoxins (especially AOH and AME) for making policies and ensuring human health.

In the last decade, liquid chromatography–tandem mass spectrometry (LC-MS/MS) (Krausová et al., 2021), immunoassay (Ackermann et al., 2011; Burkin and Kononenko, 2011; Kong et al., 2017; Man et al., 2017; Singh et al., 2018; Wang et al., 2018; Cai et al., 2021; Liang et al., 2021) and other methods with molecularly imprinted polymer or aptamers as core reagents were used to detect *Alternaria* mycotoxins in food and biology samples (Quílez-Alburquerque et al., 2021; Wang et al., 2022). Given the advantages of simple operation, less time consumption, and low cost, immunological methods were considered to be the most suitable method for the on-site detection of *Alternaria* mycotoxins in food samples (Ackermann et al., 2011), and an antibody was supposed to be the most important reagent. However, the preparation of an antibody could be influenced by many factors: it has certain randomness because of the uncertainty of the hapten design, the lack of knowledge about the recognition mechanism, and the ambiguous structure–activity relationship, which make the targeted preparation of the antibody still a considerable challenge (Liu et al., 2007). The reported immunological methods based on monoclonal antibodies (mAbs) or polyclonal antibodies (pAbs) mostly only show good recognition for a certain *Alternaria* mycotoxin. Ackermann et al. (2011) prepared mAbs and pAbs via modified AOH. The antibodies could sensitively recognize AOH but almost not recognize AME and ALT. The same result also appeared in the research of Burkin and Kononenko (2011) and Singh et al. (2018). Man et al. (2017) prepared mAbs via modified AME, which could sensitively recognize AME but almost not recognize AOH. Developing a method for simultaneous detection of *Alternaria* mycotoxins is of great significance, but antibodies for multiple recognition have not yet been developed. In our previous work, mAb, which equivalently recognizes AOH and AME, was obtained (Wang et al., 2018), and this may be a breakthrough to develop a broad-spectrum antibody for *Alternaria* mycotoxins.

With the development of bioinformatics, computer-aided and structure-based homologous modeling, molecular docking, etc. could be used to predict protein function; analyze the intermolecular interaction; and could help improve the affinity via virtual mutation calculation (Liu et al., 2021; Li et al., 2022). In this study, a hybridoma cell that secretes mAbs, which equivalently recognizes AOH and AME, was used to construct a single-chain antibody fragment (scFv) gene, and the scFv was successfully expressed. The three-dimensional structure of the scFv was constructed, the mechanism of the scFv’s equivalent recognition of AOH and AME was clarified, and the key sites of the hapten design were also discussed. It laid a theoretical foundation for further research on antibodies against *Alternaria* mycotoxins, provided a new idea for targeted preparation of antibodies that could recognize multiple hazards with similar structures and could also promote the simultaneous and rapid detection of *Alternaria* mycotoxins in food.

## Materials and Methods

### Chemicals and Reagents

AOH, AME, ALT, TeA, and tentoxin (TEN) were purchased from J&K Scientific Ltd. (Beijing, China). Hybridoma 13D8 (secretes mAb that specifically and equivalently recognizes AOH and AME) and AOH-BSA coating antigen were previously prepared in our laboratory (Wang et al., 2018). Horseradish peroxidase (HRP)-conjugated His-tag monoclonal antibody was purchased from Proteintech Group, Inc. (Rosemont, United States). The total RNA extraction kit was purchased from Qiagen (Dusseldorf, Germany) and the RT-PCR kit was purchased from ThermoFisher Scientific (Waltham, United States). The plasmid extraction kit was purchased from Tiangen Biotech Co.,, Ltd. (Beijing, China). *Escherichia coli* RV308 was purchased from ATCC. The vector pJB33 used for scFv expression was obtained as a gift from the laboratory of Andreas Plückthun (Biochemisches Institute, Universität Zürich, Switzerland). The restriction enzyme SfiI was purchased from Takara (Dalian, China). DNA polymerase and T4 DNA ligase were purchased from TransGen Biotech (Beijing, China). All the other chemical reagents were purchased from Sinopharm Chemical Reagent Co.,, Ltd. (Shanghai, China).

### Software

Discovery Studio 2019 (NeoTrident, China), GaussView 6.0 and Gaussian 09W (Gaussian, United States), ChemDraw (PerkinElmer, United States), Multiwfn 3.7 (dev) code (Lu and Chen, 2012), and VMD visualization program (Humphrey et al., 1996) were used in the study.

### Construction of Single-Chain Antibody Fragment Gene

The process of constructing the scFv was similar to the one suggested by Xu et al. (2012). Briefly, the total RNA of the hybridoma cell 13D8 was extracted, and the concentration was determined by using a Nanodrop 2000C spectrophotometer (ThermoFisher, MA, United States). Then, RNA was used as a template to synthesize cDNA through RT-PCR. The template of the cDNA and nested PCR were employed in the amplification of VH and VL. Finally, the splicing of the VL-linker–VH fragment was accomplished by SOE-PCR. The PCR product was identified by agarose gel electrophoresis, and then the gel was collected for obtaining the scFv gene fragments. The gene fragment of the scFv and pJB33 vectors was digested by SfiI, and the two digested products were linked for constructing the recombinant express vector pJB33-scFv. The recombinant vector was transformed into *E. coli* RV308 and then inoculated into the YT culture medium (containing chloramphenicol). The positive colonies were sent to Taihe Biotechnology (Beijing) Co., Ltd., for sequence identification. Details of the reaction systems are listed in the supplemental materials.

### Expression of Single-Chain Antibody Fragment

*E. coli* RV308, which contained the recombinant plasmid pJB33-scFv, was shaken at 37°C overnight. When the OD_600_ value was 0.8, 0.5 mM isopropyl-β-D-thiogalactopyranoside (IPTG) was added for induction expression (24°C, 150 rpm, 12 h). Then, ultrasound (200 W) was used to break the bacteria in an ice bath until the solution was clear and transparent, and then the solution was centrifuged at 10,000 rpm for 20 min to collect the supernatant. A Ni agarose resin column was used to purify the supernatant, then SDS-PAGE and Western blot were used for identification, and ic-ELISA was used to determine the half-maximal inhibitory concentration (IC_50_) of the scFv. The detailed steps of ic-ELISA are listed in the supplementary materials.

### Structural Analysis of Alternariol and Alternariol Monomethyl Ether

Three-dimensional structures of AOH and AME were obtained from the PubChem database and then initially prepared in GaussView 6.0. A geometry optimization procedure based on the density functional theory (DFT) was executed in the Gaussian 09W using the M06-2X density functional and TZVP basis set (Garcia et al., 2018). The hydrophobic constant (log P) was shown in ChemDraw. Solvent accessible surface areas (SASA) were extracted by VMD visualization program. The molecular electrostatic potential (ESP)-mapped van der Waals surface was rendered by means of VMD based on the files exported by Multiwfn (Wen et al., 2020). Molecular structural similarity was calculated by “Molecular Overlay” in Discovery Studio 2019 (DS).

### Homology Modeling of Single-Chain Antibody Fragment

The spatial model of the scFv was constructed on the basis of the PDB database. First, the complementarity-determining regions (CDRs) of VH and VL were annotated by the “Model Antibody” module in DS based on IMGT. The sequence with the highest similarity and identity was selected as the template to generate scFv spatial models. According to the value of the probability density function (PDF) (automatically generated by DS), the best simulated conformation was selected, then the CDRs of the model were optimized by BLAST to form the final model. The Ramachandran plot diagram and profile-3D score were used to evaluate the final spatial mode.

### Molecular Docking and Dynamic Simulation

AOH and AME were docked into the active pocket of the scFv by the “CDOCKER Module” of DS after being drawn by the “Sketch Module” and given the CHARMm force field for structure optimization (Zhang et al., 2019). The active pocket was defined as all atoms within a 10 Å radius of the protein cavity. All water molecules were removed, and hydrogen atoms were added in. The remaining parameters were set to the default values of the program. The AOH–scFv and AME–scFv complexes were given the CHARMm36 force field and explicit periodic boundary solvation. The molecular dynamic simulation was carried out via the “Standard Dynamics Cascade” module of DS.

### Virtual Mutation

The complex was given the CHARMm force field, and all amino acids within a 3 Å radius of the binding site were subjected to single-point saturation mutation in the “Design protein” module of DS. When setting parameters, pH and ionic strength were set to 7.45 and 0.15 M, respectively, and the amino acid causing scFv affinity to increase or decrease was predicted according to the mutation results. A mutation energy less than −0.5 could lead to an increase in the affinity, a mutation energy between −0.5 and 0.5 had no significant effect on the affinity, while a mutation energy greater than 0.5 could lead to a decrease in the affinity.

## Results and Discussion

### Preparation of Single-Chain Antibody Fragment

The smallest immunoglobulin antigen-binding fragment that keeps a complete antigen-binding site was the Fv fragment, which only consists of a variable (V) region. Then, a soluble and flexible peptide linker was used to stabilize the connection of the V region to form an scFv (Bird et al., 1988). Even though increasingly recombinant production systems have been developed (such as *E. coli*, yeast, insect cell lines, mammalian cells), *E. coli* is still the most commonly used system for the scFv. In this study, *E. coli* RV308 could express soluble scFv, and the scFv could enter the periplasmic cavity under the action of a guiding peptide and fold correctly under the action of an oxidizing environment and various molecular partners. A peptide linker (G_4_S)_3_ could provide enough flexibility for VH and VL domains, so it could form monovalent antigen-binding sites equivalent to Fab fragments of the mAb (Huston et al., 1991).

The concentration of the total RNA was 536.2 ng/μL, and the A260/A280 value was 2.12, which indicated that there was no contamination. As shown in Supplementary Figure S1, agarose gel electrophoresis showed that the VH was about 360 bp, VL was about 320 bp, and the scFv was about 750 bp. The full-length fragment of the scFv gene was successfully constructed. SDS-PAGE and Western blot results are shown in Supplementary Figure S2. All the samples showed a single band at about 30 kDa, indicating that there were no other miscellaneous proteins, and the scFv was successfully expressed. The concentration of the scFv was 1.4 mg/ml. The IC_50_ values of the scFv for AOH and AME were 509.0 ng/ml and 565.7 ng/ml, respectively (Supplementary Figure S3), and there is no cross-recognition for ALT, TeA, and TEN. It proved that the expressed scFv had biological activity. Due to the lack of structure, the affinity of the scFv was lower than that of the parent mAb. The same results also appeared in other research studies (KorpimäKi et al., 2002; Xie et al., 2020). Although the affinity decreased, the recognition of AOH, AME, ALT, TeA, and TEN was consistent with the parent antibody, which proved the reliability of the results.

### Structural Analysis of Alternariol and Alternariol Monomethyl Ether

**FIGURE 2:**
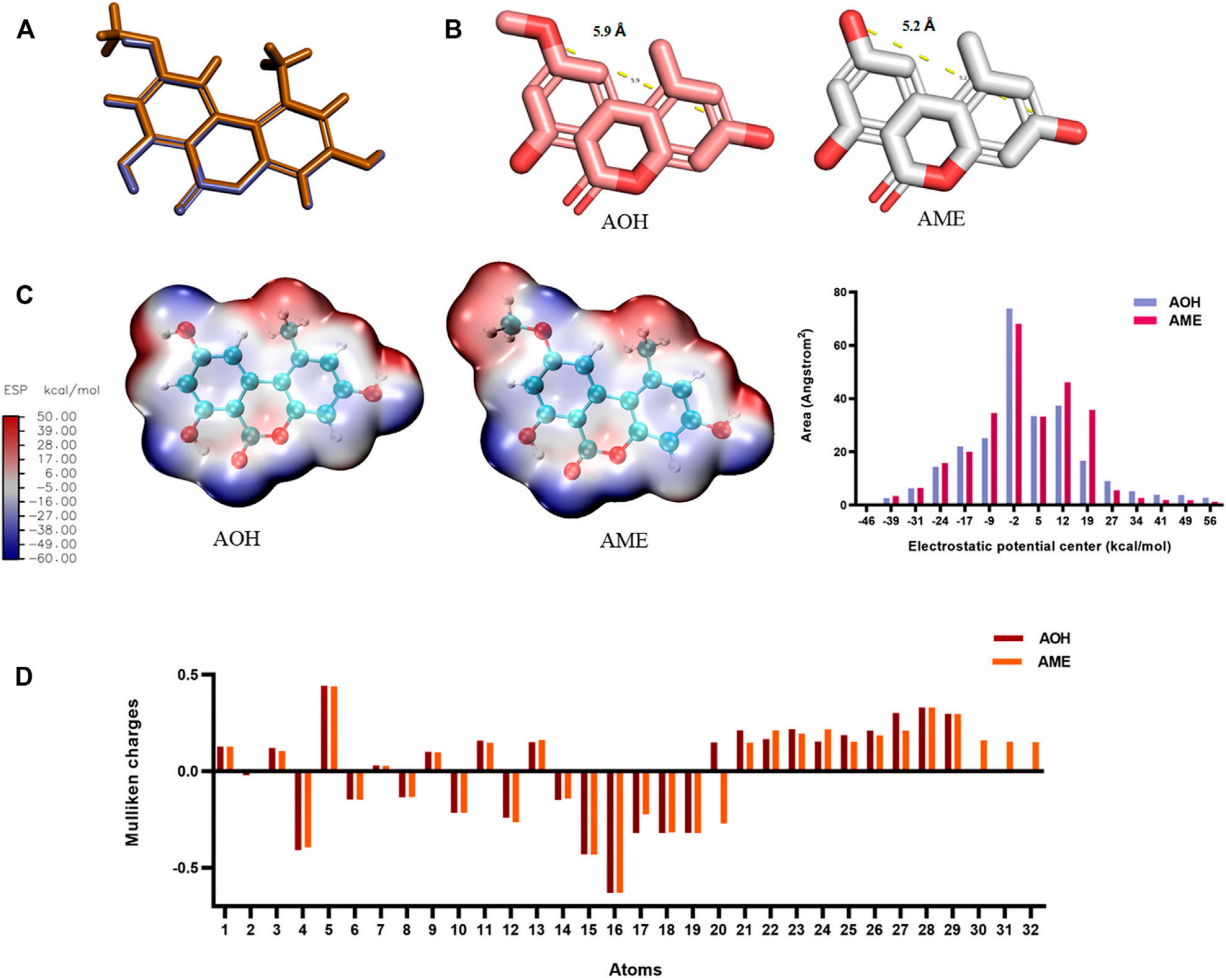
Structural analysis of AOH and AME. **(A)** Molecular overlay (purple represents AOH and yellow represents AME). **(B)** Molecular scale of AOH and AME. **(C)** ESP of AOH and AME. **(D)** Mulliken charge of AOH and AME.

As shown in Figure 2A, the molecular overlay was based on the influence of 50% of the space field and 50% of the electrostatic field. The coincidence degree of AOH and AME reached 98.76%, and there was difference in the C9 group, while the coincidence degree of AOH with ALT, TeA, and TEN was 79.53%, 61.89%, and 68.43%, respectively. As shown in Figure 2B, the molecular scales of AOH and AME were calculated, the distance between the C3 hydroxyl group and C9 hydroxyl group of AOH was 5.2 Å, and the distance between the C3 hydroxyl group and C9 methoxy group of AME was 5.9 Å. The difference was negligible. Figure 2C shows that the methoxy group was positively charged, while the hydroxyl group was negatively charged. If C9 was the antigen-recognition site, it might greatly affect the recognition result of the antibody. Figure 2D shows the Mulliken charge of different atoms of AOH and AME (Supplementary Figure S4), with the largest charge difference of numbers 20, 30, 31, and 32, pointing to C9 methoxy of AME. In addition, log P and SASA of AOH and AME were 2.48 and 443.54, and 2.74 and 463.65, respectively, which indicated that AOH and AME had very similar hydrophobic properties. The aforementioned results provided necessary conditions for the appearance of antibody equivalent recognition.

### Construction of Single-Chain Antibody Fragment 3D Structure

**FIGURE 3:**
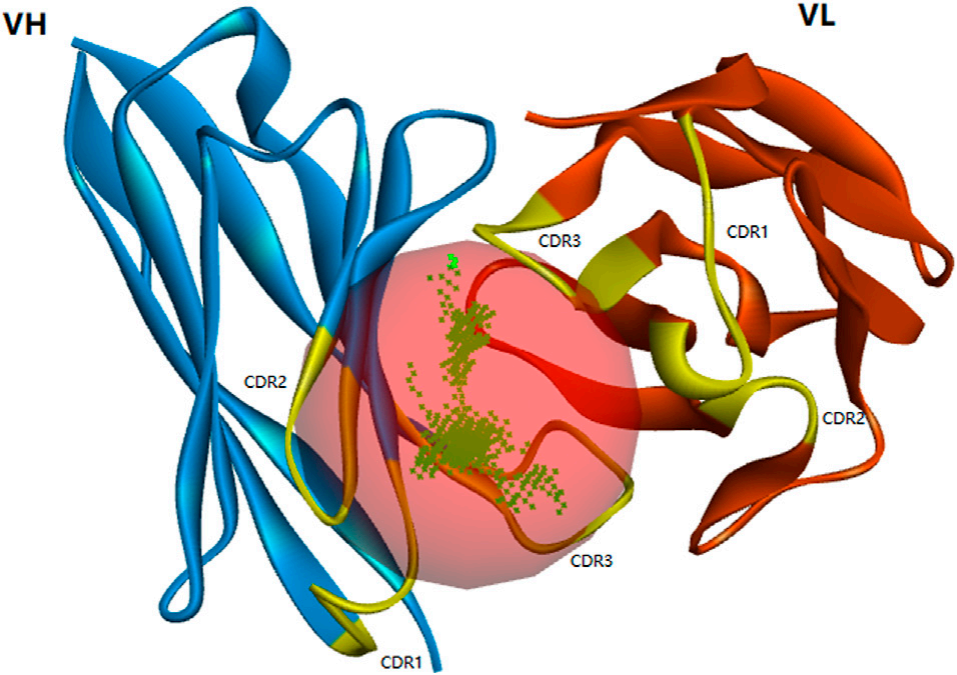
Optimized spatial structure model of scFv (yellow highlights represent the distribution of CDR regions and red balls represent active cavity located in CDR regions).

X-ray, nuclear magnetic resonance (NMR), and cryo-electron microscopy are commonly used to directly obtain the 3D structure of proteins, but these methods are time-consuming and expensive. Homologous modeling has been widely used (Wang et al., 2020). As shown in Supplementary Figure S5, three complementarity-determining regions (CDRs) were annotated in VH and VL, respectively. Blue represented the VL region, and the CDRs were located at 27–33 (CDR1), 51–53 (CDR2), and 89–97 (CDR3), respectively. Green represented the VH region, and the CDRs were located at 153–160 (CDR1), 178–184 (CDR2), and 223–236 (CDR3), respectively. The template 6BDZ with 95% similarity and 87.8% identity was selected from the PDB database, and then the best model with the smallest PDF value was constructed (Figure 3). It showed that CDR1, CDR2, and CDR3 of the heavy chain and CDR3 of the light chain formed the binding active cavity.

A reliable model should have more than 90% residues in the allowable area of the Ramachandran plot (Zhang et al., 2019). As shown in Supplementary Figure S6A, the blue and purple regions represent the allowed regions, and red represents the disallowed ones. In this model, 99.5% of amino acid residues were located in the allowed regions, while 0.5% of amino acids were distributed in the disallowed regions. The profile-3D evaluation of amino acid sequence-matching degree showed that no amino acids were in the low sub-region structure and mismatched the sequence (Supplementary Figure S6B), indicating that the scFv 3D model was reliable.

### Molecular Docking and Dynamic Simulation

**FIGURE 4:**
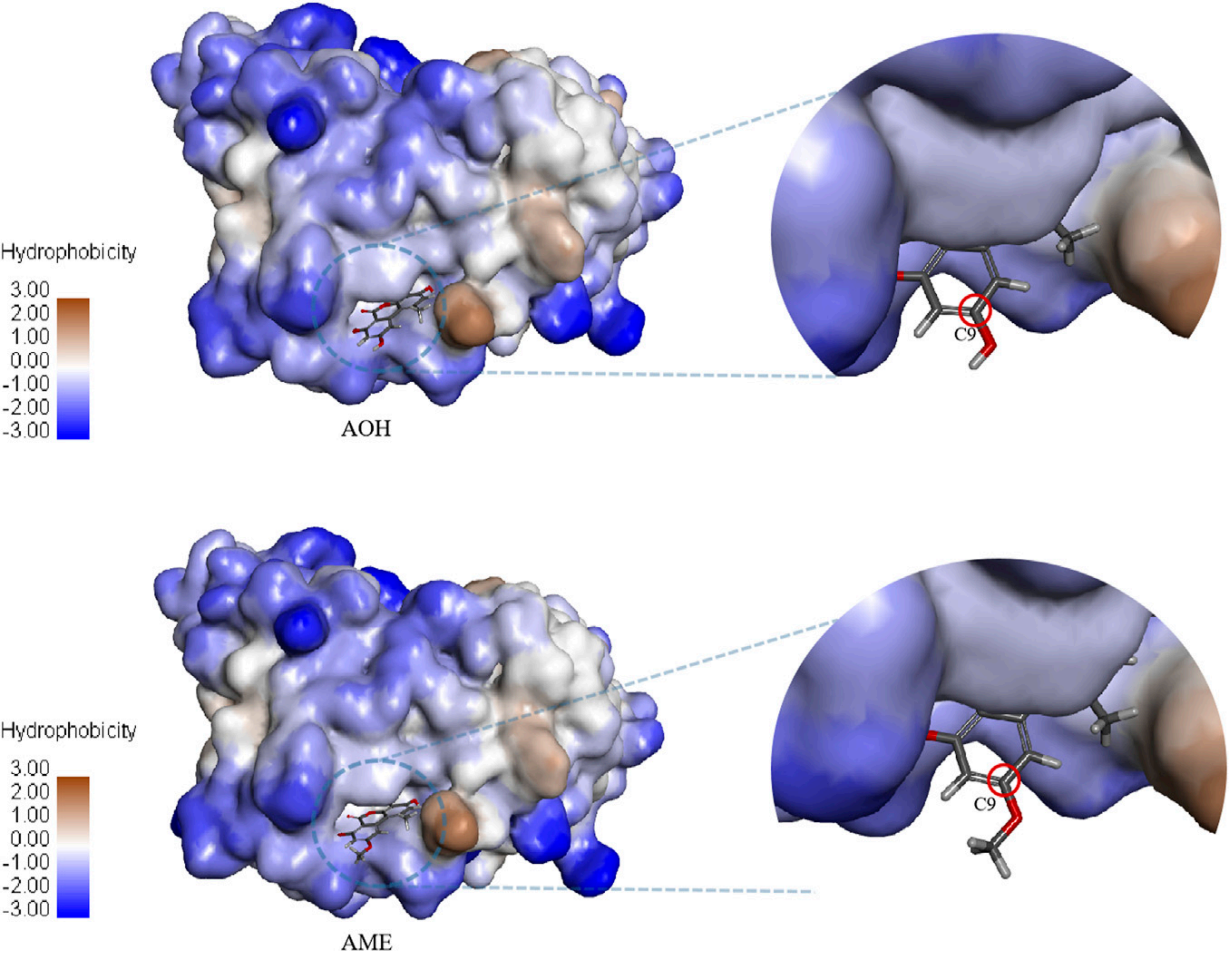
Molecular docking model of AOH/AME and scFv.

**FIGURE 5:**
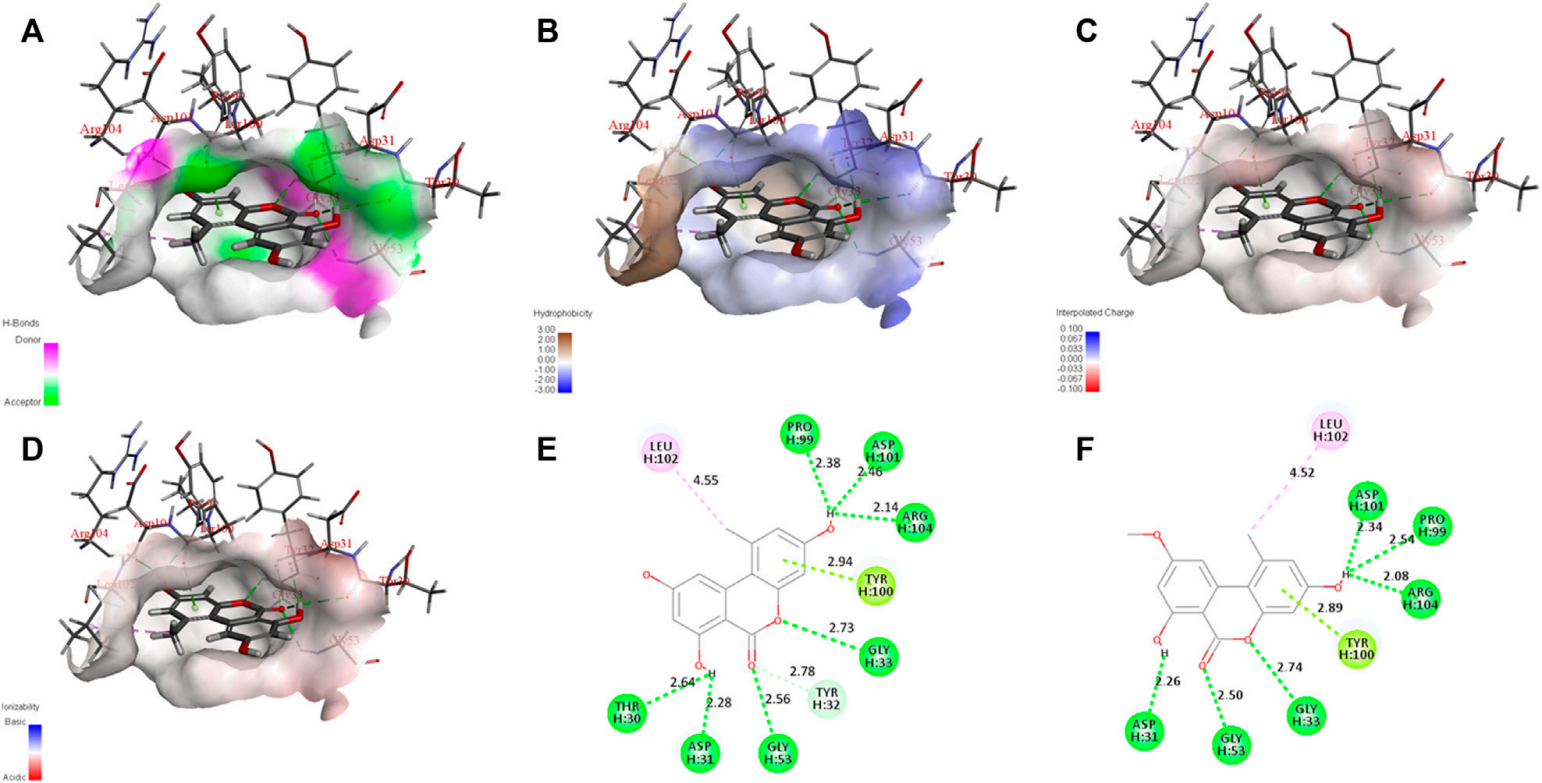
Interaction between AOH/AME and scFv. **(A)** Hydrogen bond donors and acceptors around the binding cavity (green represents the receptor and purple represents the donor.) **(B)** Hydrophobicity around the binding cavity (blue represents hydrophilicity and brown represents hydrophobicity). **(C)** Charge around the binding cavity (blue represents positive electricity and red represents negative electricity). **(D)** The acid–base around the binding cavity (blue represents basic and red represents acidic). **(E)** Two-dimensional diagram of interactions between scFv and AOH. **(F)** Two-dimensional diagram of interactions between scFv and AME (green represents hydrogen bond interaction, cyan represents π-lone pair interaction, and pink represents alkyl interaction).

AOH, AME, and the scFv were subjected to semiflexible docking by CDOCKER (Zhang et al., 2019). As shown in Figure 4, AOH and AME entered the binding site of the scFv vertically, the whole parent nucleus was surrounded by the cavity, and C3 hydroxyl was deeply inside to the cavity, while C9 hydroxyl was exposed outside adequately. The donor and acceptor of the hydrogen bond, hydrophobicity, charge, and acid–base around the binding cavity are shown in Figure 5. Figures 5A–D show that there are abundant hydrogen-bond donors and acceptors around the binding cavity, while AOH and AME have multiple hydroxyl and oxygen atoms, which provides favorable conditions for the formation of a hydrogen bond between AOH/AME and the scFv. The hydrophobicity of the binding cavity is almost weak, while AOH and AME have multiple benzene ring structures, which may be one of the reasons for the undesirable affinity of antibodies. Moreover, the cavity was a neutral environment; thus, it is difficult to form an electrostatic interaction between AOH/AME and the scFv.

In Figure 5E, the molecular docking results show that 10 amino acids interacted with AOH; Thr 30 (2.64 Å) and Asp 31 (2.28 Å) formed hydrogen bonds with C7 hydroxyl groups; Gly 53 (2.56 Å) and Tyr 32 (2.78 Å) formed hydrogen bonds with C6 oxygen; Gly 33 (2.73 Å) formed hydrogen bonds with O5; Tyr 100 (2.94 Å) formed π-lone pair interaction with a benzene ring; Pro 99 (2.38 Å), Asp 101 (2.46 Å), and Arg 104 (2.14 Å) formed hydrogen bonds with C3 hydroxyl groups; and Leu 102 (4.55 Å) formed an alkyl interaction with C1 methyl groups. As shown in Figure 5F, eight amino acids interacted with AME; Asp 31 (2.26 Å) formed hydrogen bonds with C7 hydroxyl groups; Gly 53 (2.50 Å) formed hydrogen bonds with C6 oxygen, Gly 33 (2.74 Å) formed hydrogen bonds with O5; Tyr 100 (2.89 Å) formed π-lone pair interaction with a benzene ring; Pro 99 (2.54 Å), Asp 101 (2.34 Å), and Arg 104 (2.08 Å) formed hydrogen bonds with C3 hydroxyl groups; and Leu 102 (4.52 Å) formed alkyl interaction with C1 methyl. These results showed that the interaction between AOH, AME, and the scFv was mainly of hydrogen bonds. The amino acids that produce the interaction between them were the same, and this was the intuitive reason for the equivalent recognition of the antibody. The structural difference between AOH and AME was concentrated at the C9 site; however, in the docking model, the C9 sites of both AOH and AME were exposed outside the binding cavity and have no relevant force with the scFv, so the influence of structural differences on antibody recognition could be ignored, and this may be the most important factor of equivalent recognition.

As shown in Table 1, the mAb and pAb obtained by mixing AOH C3 and C7 hydroxyl-modified immunogens could only specifically recognize AOH (Ackermann, et al., 2011; Kong et al., 2017) and the mAb obtained by the AME C3 hydroxyl-modified immunogens could only recognize AME (Man et al., 2017). Wang et al., 2018 obtained mAbs that could specifically and equivalently recognize AOH and AME through mixed immunogens modified by C3, C7, and C9 hydroxyl groups of AOH. It is speculated that modifying the C3 and C7 hydroxyl will expose the structural difference, so the obtained antibodies could recognize certain compounds (Ackermann, et al., 2011). However, the structural difference between AOH and AME will be well-solved by modifying the C9 site; therefore, the modification of the C9 site may be the key point to the targeted preparation of antibodies that recognize AOH and AME equivalently. In the process of trying to dock ALT with the scFv model, it was shown that effective docking could not be formed, which was consistent with the conclusion that the antibody had no cross-reaction to ALT, which further demonstrated the reliability of the docking model. The failure to recognize ALT may be due to the addition of the methyl structure, which caused C3 hydroxyl to fail to enter the binding cavity. Thus, in the process of immunogen modification, exposing the common structure of a class of structural analogs and avoiding structural differences between the immunogen and target compounds may be a possible way to solve the difficulty of preparing broad-spectrum antibodies.

**TABLE 1:** Existing methods for preparing antibodies for AOH and AME.

Antibody type	Compound	Modification method	Modification site	Cross-reaction rate (%)	
mAb	AOH	Mannich	C3 or C7 hydroxyl	AOH (100)	Ackermann, et al. (2011)
				AME (0.9)	
pAb	AOH			AOH (100)	
				AME (<0.5)	
				ALT (<0.5)	
mAb	AME	Carboxyl derivative modification	C3 hydroxyl	AME (100)	Man et al. (2017)
				AOH (2.9)	
mAb	AOH	CDI	C3 or C7 or C9 hydroxyl	AOH (100)	Kong et al. (2017)
				AME (24.6)	
mAb	AOH	Alkylation reaction	C3 or C7 or C9 hydroxyl	AOH (100)	Wang et al. (2018)
				AME (97)	

The results of molecular docking provided an explanation for the state of antigen–antibody interaction, but in fact, antigen–antibody interaction was a dynamic reaction process. Molecular dynamics is a technique for studying the physical motion of atoms or molecules in a complex system. It could predict the stability of the complex and is performed to analyze the stability of the protein–ligand complexes on a picosecond (ps) scale. The movement of the atoms in a chemical complex is discovered by keeping the temperature, volume, and pressure parameters constant for a set period of time (Bhardwaj et al., 2022). In this study, the dynamic interaction process of AOH–scFv and AME–scFv complexes in an aqueous solvent was studied. In 200 ps, the C9 site of AOH and AME was always exposed outside the binding cavity, which further confirmed the conclusion that the modification of the C9 site was the key point for preparing an antibody that recognizes AOH and AME equivalently.

### Affinity Evolution

The scFv provides conditions for affinity evolution *in vitro.* Xie et al. (2020) explored the recognition mechanisms between amantadine and the scFv, and after virtual mutation and expression, the results indicated that the sensitivity of the scFv was improved by 3.9 times by mutating Gly 107 to Phe. In this study, there were 13 amino acids (Thr 30, Asp 31, Tyr 32, Gly 33, Trp 52, Gly 53, Val 98, Pro 99, Tyr 100, Asp 101, Leu 102, Arg 104, and Tyr 105 of VH) within a 3 Å radius of the binding site. As shown in Supplementary Table S5, the mutations of Thr 30, Asp 31, Pro 99, Leu 102, and Arg 104 almost will not affect the affinity of the scFv, whereas mutations of Gly 33 to Leu and Tyr 100 to Phe could improve the affinity of the scFv. In addition, the mutation of Tyr 32, Gly 33, Trp 52, Gly 53, Val 98, Tyr 100, Asp 101, and Tyr 105 could all reduce the affinity of the scFv. These results provided a scheme for further enhancing the affinity of the scFv for the detection of actual samples.

## Conclusion

In summary, this study expressed an scFv that could equivalently recognize AOH and AME and laid a foundation for the directed evolution of an antibody at the molecular level. It could promote the development of rapid detection technology for both AOH and AME in food. By analyzing the molecular structure and interaction processes, it was clarified that the equivalent recognition depended on the structural similarity of AOH and AME, and almost the same interaction between the amino acid and acting force was found. The structural difference was well-solved because different C9 sites were exposed outside the binding cavity, which made the common structure of AOH and AME fully exposed to the antibody. Hence, C9 was considered to be the decisive site to determine the equivalent recognition of the antibody in the process of hapten modification. Therefore, the exposure of common structures and avoiding re-formation of similar structures may be an important strategy for the targeted preparation of antibodies that recognize multiple hazards with similar structures, which could guide future hapten design and antibody improvement.

## Data Availability

The datasets presented in this study can be found in online repositories. The names of the repository/repositories and accession number(s) can be found in the Supplementary Material.
