# Supplementary material for: Recombinant Antibody-Based and Computer-Aided Comprehensive Analysis of Antibody’s Equivalent Recognition Mechanism of Alternariol and Alternariol Monomethyl Ether
Source: Front Chem. 2022 Apr 14;10:871659. doi: 10.3389/fchem.2022.871659 (PMC9046909; doi:10.3389/fchem.2022.871659)
Supplement: Supplementary file 1 [file DataSheet1.docx]

Supplementary Material

**PCR primer**

scFv-VL-F1: GATGTTGTGATGACCCAAACT

scFv-VL-R1: TTTTATTTCCAGCTTGGTCCC

scFv-VL-F2: ctacagcaggcccagccggccatggcgGATGTTGTGATGACCCAAACT

scFv-VL-R2: ggagccgccgccgccagaaccaccaccaccagaaccaccaccaccTTTTATTTCCAG

CTTGGTCCC

scFv-VH-F1: CAGGTCCAACTGCAGCAACC

scFv-VH-R1: TGAGGAGACTGTGAGAGTGG

scFv-VH-F2: ggcggcggcggctccggtggtggtggatccCAGGTCCAACTGCAGCAACC

scFv-VH-R2: TCGCTAATCAGTTTTTGTTCGGCGGCCGCTGAGGAGACTGTG

AGAGTGG

scFv-VH-R3: cggagtcaggcccccgaggccAGGTCTTCTTCGCTAATCAGTTTT

The sequence of primers were all from 5'-terminal to 3'-terminal, the yellow highlights were the restriction site of Sfi I, and the gray highlights were linker peptide (G_4_S)_3_, its 5'-terminal was complementary to the 3'-terminal of VH fragment and 3'-terminal was complementary to the 5'-terminal of VL fragment.

**Extraction of total RNA**

Firstly when hybridoma cell was in logarithmic growth, cleaned the surface of cell culture plate with sterile PBS. Secondly, added 1 mL lysate, blew, sucked and mixed evenly, then added the lysate to QIAshredder centrifugal column, centrifuged at 10,000 rpm for 2 min. Thirdly, added the upper liquid into another centrifuge tube, centrifuged at 10,000 rpm for 2 min, collected the upper liquid. Fourthly, added equal volume of 70% ethanol into the centrifuge tube, blew, sucked and mixed evenly, then added the liquid into RNeasy centrifuge column, centrifuged at 10,000 rpm for 15 s. Fifthly, added 350 μL RW1 solution, centrifuged at 10,000 rpm for 15 s, then added 500 μL RPE solution, centrifuged at 10,000 rpm for 15 s. Finally added 30 μL RNase-free water, centrifuged at 10,000 rpm for 1 min, and collected the solution. The used solution and centrifugal column were all provided by the QIAGEN RNeasy mini kit.

**Synthesis of cDNA and cDNA-AS-Linker**

Total RNA was used as template, then cDNA was synthesized by SuperScript^TM^ First-Strand Synthesis System for RT-PCR kit. Firstly, 5 μg of RNA, 1 μL of 50 μM Oligo (dT), 1 μL of 10 mM dNTPmix were mixed then diethyl pyrocarbonate (DEPC) water was added to make the total volume was 10 μL. The mixture was heated at 65°C for 5 min and inserted into ice for 2 min. Secondly, added the mixture into reaction system A (Table S1)and incubated in 50°C water bath for 50 min, then heated the mixture at 85 °C for 5 min and inserted the test tube into ice to terminate the reaction. Thirdly, 1 μL of RNase H was added into the mixture then incubated at 37°C for 20 min to obtain cDNA. Fourthly, incubated the reaction system B (Table S1) at 22°C for 16 h. Finally, 38 μL of 10 mM Tris-HCl (pH 8.0) and 2 μL of 0.5 M EDTA (pH 8.0) were added to stop the reaction. The product cDNA-AS-Linker was stored at -20℃.

**Cloning and amplification of VH and VL gene**

The first round of amplification used cDNA-AS-Linker as template and the reaction was carried out with reaction system 1 (Table S2), AS04-primer and IgG_2b_ primer were used as primers to preliminarily amplify VH gene, AS04-primer and Kappa primer were used as primers to preliminarily amplify VL gene. After the reaction, the product was identified by agarose gel electrophoresis, and the target fragment was recovered. VH and VL gene amplified in the first round was used as templates in nested PCR of reaction system 2 (Table S2), scFv-VH-F1, scFv-VH-R1 were used as primers in the second round, scFv-VH-F2, scFv-VH-R2 were used as primers in the third round and scFv-VH-F2, scFv-VH-R3 were used as primers in the fourth round to amplify VH. scFv-VL-F1, scFv-VL-R1 were used as primers in the second round, scFv-VL-F2, scFv-VL-R2 were used as primers in the third round to amplify VL. PCR reaction conditions were as follows: pre-denaturation at 95℃ for 5 min then 95℃ for 20 s, 55℃ for 20 s and 72℃ for 2 min for 30 cycles in total, then extended at 72℃ for 10 min at the end. After the reaction, the products were identified by agarose gel electrophoresis, and the target fragments were recovered to obtain VH and VL gene.

**Splicing and amplification of scFv gene**

Mixed the reaction system (Table S3) evenly, then centrifuged briefly. The PCR reaction conditions were as follows: pre-denaturation at 95℃ for 5 min then 95℃ for 1 min, 55℃ for 2 min and 72℃ for 2 min for 30 cycles in total, extended at 72℃ for 10 min at the end. After the reaction, the scFv gene (VL-Linker-VH) was identified by agarose gel electrophoresis, and the target fragment was recovered.

**Construction of recombinant expression vector**

The enzyme digestion of scFv gene was carried out in reaction system A (Table S4), pJB33 expression vector was carried out in reaction system B (Table S4) and the construction of scFv-pJB33 expression vector was carried out in reaction system C (Table S4). After reacted at 50°C for 16 h, the product was identified by agarose gel electrophoresis, and the target fragment was recovered.

**Transformation of recombinant plasmid**

Ten microliters of scFv-pJB33 ligation product was dialyzed with pure water for 30 min. The competent cells RV308 were taken out from the ultra-low temperature refrigerator at -80℃ and melted on ice. The dialysis product was added into 50 μL of competent cells of RV308, transferred them into a clean, dry and precooled transformation cup then conducted electric shock transformation. Conditions: voltage 2.5 kV, resistance 150 Ω, capacitance 20 μF for 5 ms. After electrotransfer, 800 μL of 2×YT culture medium was added to resuspend the cells immediately, and the cells were cultured at 37°C then shocked at 250 rpm for 1 h. Then 100 μL bacterial solution was coated on a 2×YT plate containing chloramphenicol, inverted overnight at 37℃.

**ic-ELISA step of scFv**

Firstly, diluted the coating antigen AOH-BSA with coating buffer, 100 μL per well, incubated at 4℃ for 16 h, then washed for 3 times. Secondly, added 150 μL of blocking buffer per well, incubated at 37°C for 2 h, washed for 3 times. Thirdly, added 50 μL of standard solution per well, then added equal volume of scFv solution, incubated at 37°C for 30 min, washed for 3 times. Fourthly, added 100 μL of HRP-conjugated His-Tag antibody per well, incubated at 37°C for 30 min, washed for 3 times. Finally, added 100 μL of coloring solution (TMB and H_2_O_2_), incubated at 37°C for 15 min；termination then added 50 μL of H_2_SO_4_ to stop the reaction. The OD_450nm_ value of was determined by microplate reader (Multiskan FC, Thermo, China).

**Supplementary Table 1.** Composition of cDNA reaction system. (A) Synthesis of cDNA. (B) Synthesis of cDNA-AS-Linker.

| Reaction system | Component | Volume  (μL) |
| --- | --- | --- |
| A | 10× RT Buffer | 2 |
|  | 25 mM MgCl_2_ | 4 |
|  | 0.1 M DTT | 2 |
|  | 40 U/μL RNase OUT | 1 |
|  | 200 U/μL Super Script III RT | 1 |
| B | cDNA | 2 |
|  | 10× reaction buffer | 1 |
|  | 50% PEG 8000 | 5 |
|  | T4 RNA ligase | 1 |
|  | 10 mM ATP | 1 |
|  | AS-Linker | 1 |

**Supplementary Table 2.** Composition of cloning and amplification of VH and VL genes reaction system. (A) Preliminary amplification. (B) Nested PCR.

| Reaction system | Component | Volume (μL) |
| --- | --- | --- |
| A | cDNA-AS-Linker | 1 |
|  | 2× Ex taq mix | 10 |
|  | AS04-primer | 1.5 |
|  | IgG_2b_ primer/Kappa primer | 1.5 |
|  | sterile water | 6 |
| B | 2× Ex taq mix | 10 |
|  | PCR products | 1 |
|  | IgG_2b_ primer | 2 |
|  | Kappa primer | 2 |
|  | sterile water | 5 |

**Supplementary Table 3.** Composition of scFv splicing and amplification system.

| Component | Volume (μL) |
| --- | --- |
| VH | 0.5 |
| VL | 0.5 |
| VL-F2 | 1 |
| VH-R3 | 1 |
| 2×Ex taq mix | 10 |
| sterile water | 7 |

**Supplementary Table 4.** Composition of enzyme digestion and constructing scFv-pJB33 expression vector system. (A) Enzyme digestion of scFv. (B) Enzyme digestion of pJB33. (C) Constructing of scFv-pJB33.

| Reaction system | Component | Volume (μL) |
| --- | --- | --- |
| A | scFv | 2 |
|  | 10× CutSmart buffer | 5 |
|  | Sfi I | 1 |
|  | 100× BSA solution | 0.5 |
|  | sterile water | 30.5 |
| B | pJB33 | 2 |
|  | 10× CutSmart buffer | 5 |
|  | Sfi I | 1 |
|  | 100× BSA solution | 0.5 |
|  | sterile water | 30.5 |
| C | scFv | 1.8 |
|  | pJB33 | 0.7 |
|  | 10× enzyme digestion buffer | 1 |
|  | T4 RNA ligase | 1 |
|  | sterile water | 6.5 |

**Supplementary Table 5.** The result of virtual mutation.

| Mutation | Mutation Energy  (kcal/mol) | Effect | Mutation | Mutation Energy  (kcal/mol) | Effect | Mutation | Mutation Energy  (kcal/mol) | Effect | Mutation | Mutation Energy  (kcal/mol) | Effect |
| --- | --- | --- | --- | --- | --- | --- | --- | --- | --- | --- | --- |
| H:GLY33>LEU | -0.56 | Stabilizing | H:TRP52>GLU | 1.61 | Destabilizing | H:GLY53>LYS | 6.46 | Destabilizing | H:ASP101>LYS | 0.61 | Destabilizing |
| H:TYR100>PHE | -0.97 | Stabilizing | H:TRP52>GLY | 1.64 | Destabilizing | H:GLY53>MET | 5.15 | Destabilizing | H:ASP101>THR | 0.63 | Destabilizing |
| H:ASP31>TYR | 0.66 | Destabilizing | H:TRP52>HIS | 0.53 | Destabilizing | H:GLY53>PHE | 1.02 | Destabilizing | H:ASP101>VAL | 0.7 | Destabilizing |
| H:GLY33>ALA | 0.88 | Destabilizing | H:TRP52>ILE | 1.07 | Destabilizing | H:GLY53>PRO | 4.72 | Destabilizing | H:ARG104>TYR | 0.57 | Destabilizing |
| H:GLY33>ASN | 0.97 | Destabilizing | H:TRP52>LEU | 1.05 | Destabilizing | H:GLY53>SER | 1.39 | Destabilizing | H:TYR105>ALA | 0.99 | Destabilizing |
| H:GLY33>ASP | 1.71 | Destabilizing | H:TRP52>LYS | 1.56 | Destabilizing | H:GLY53>THR | 3.73 | Destabilizing | H:TYR105>ARG | 0.75 | Destabilizing |
| H:GLY33>CYS | 0.6 | Destabilizing | H:TRP52>MET | 1.35 | Destabilizing | H:GLY53>TRP | 1.18 | Destabilizing | H:TYR105>ASN | 1.17 | Destabilizing |
| H:GLY33>GLU | 0.97 | Destabilizing | H:TRP52>PHE | 1.47 | Destabilizing | H:GLY53>TYR | 1.17 | Destabilizing | H:TYR105>ASP | 1.85 | Destabilizing |
| H:GLY33>GLY | 0.55 | Destabilizing | H:TRP52>PRO | 1.55 | Destabilizing | H:VAL98>ARG | 0.91 | Destabilizing | H:TYR105>CYS | 0.84 | Destabilizing |
| H:GLY33>HIS | 1.54 | Destabilizing | H:TRP52>SER | 1.59 | Destabilizing | H:VAL98>ASP | 0.67 | Destabilizing | H:TYR105>GLN | 0.93 | Destabilizing |
| H:GLY33>ILE | 0.84 | Destabilizing | H:TRP52>THR | 1.42 | Destabilizing | H:VAL98>GLN | 0.7 | Destabilizing | H:TYR105>GLU | 1.19 | Destabilizing |
| H:GLY33>LYS | 1.64 | Destabilizing | H:TRP52>TYR | 0.87 | Destabilizing | H:VAL98>HIS | 0.68 | Destabilizing | H:TYR105>GLY | 1.08 | Destabilizing |
| H:GLY33>MET | 1.8 | Destabilizing | H:GLY53>ALA | 1.54 | Destabilizing | H:VAL98>LYS | 0.59 | Destabilizing | H:TYR105>ILE | 0.62 | Destabilizing |
| H:GLY33>PRO | 1.68 | Destabilizing | H:GLY53>ARG | 5.17 | Destabilizing | H:VAL98>PHE | 4.29 | Destabilizing | H:TYR105>LEU | 0.66 | Destabilizing |
| H:GLY33>TYR | 4.17 | Destabilizing | H:GLY53>ASN | 4.75 | Destabilizing | H:VAL98>TRP | 5.15 | Destabilizing | H:TYR105>LYS | 0.69 | Destabilizing |
| H:GLY33>VAL | 0.99 | Destabilizing | H:GLY53>ASP | 5.45 | Destabilizing | H:VAL98>TYR | 3.45 | Destabilizing | H:TYR105>MET | 0.56 | Destabilizing |
| H:TRP52>ALA | 1.56 | Destabilizing | H:GLY53>CYS | 1.4 | Destabilizing | H:PRO99>LYS | 0.5 | Destabilizing | H:TYR105>PHE | 0.69 | Destabilizing |
| H:TRP52>ARG | 1.32 | Destabilizing | H:GLY53>GLN | 4.35 | Destabilizing | H:TYR100>ALA | 0.57 | Destabilizing | H:TYR105>PRO | 0.95 | Destabilizing |
| H:TRP52>ASN | 0.68 | Destabilizing | H:GLY53>GLU | 2.89 | Destabilizing | H:TYR100>GLY | 0.67 | Destabilizing | H:TYR105>SER | 1.05 | Destabilizing |
| H:TRP52>ASP | 0.98 | Destabilizing | H:GLY53>HIS | 1.23 | Destabilizing | H:TYR100>LYS | 0.56 | Destabilizing | H:TYR105>THR | 0.92 | Destabilizing |
| H:TRP52>CYS | 1.3 | Destabilizing | H:GLY53>ILE | 6.08 | Destabilizing | H:TYR100>TRP | 0.55 | Destabilizing | H:TYR105>VAL | 0.8 | Destabilizing |
| H:TRP52>GLN | 1.6 | Destabilizing | H:GLY53>LEU | 2.36 | Destabilizing | H:ASP101>HIS | 0.63 | Destabilizing |  |  |  |

**Supplementary Figure 1.** Agarose gel electrophoresis of VH, VL and scFv gene fragments.


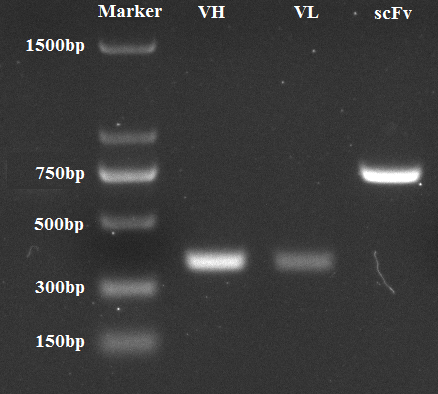


**Supplementary Figure 2.** SDS-PAGE and Western blot results of scFv. (A) SDS-PAGE. (B) Western blot.


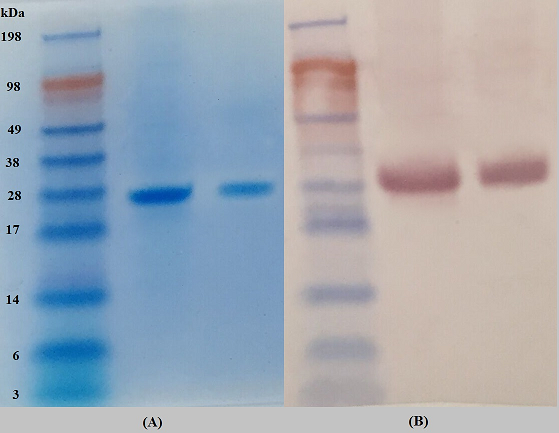


**Supplementary Figure 3.** ELISA standard curves of scFv.





**Supplementary Figure 4.** Atomic serial number and distribution of AOH and AME.


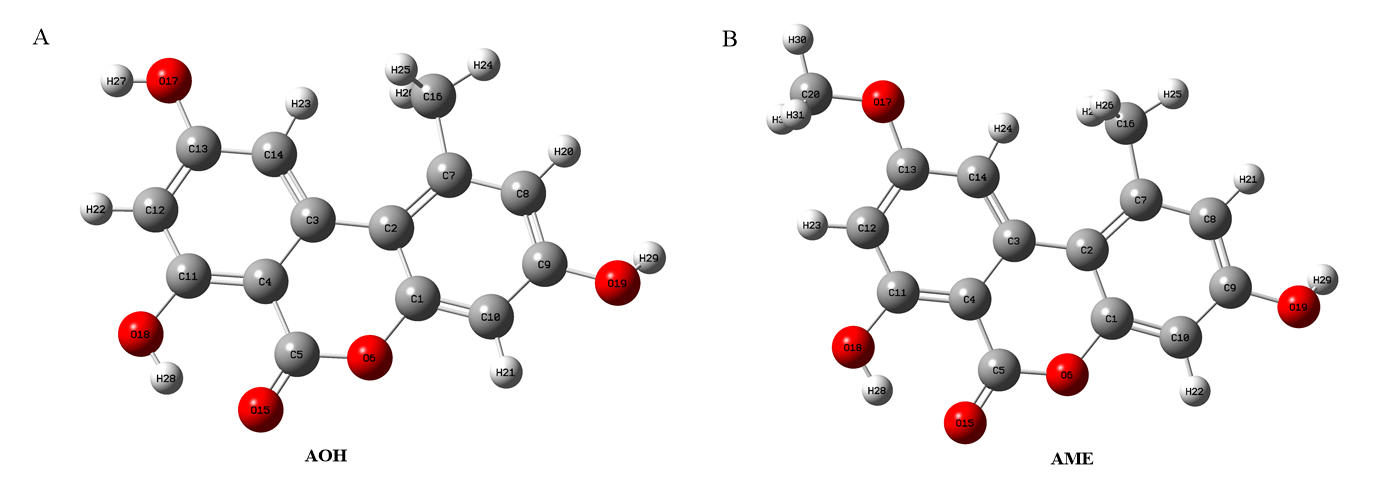


**Supplementary Figure 5.** Distribution of CDR regions. (A) Distribution of CDR regions of scFv. (B) Distribution of CDR regions of VH. (C) Distribution of CDR regions of VL.


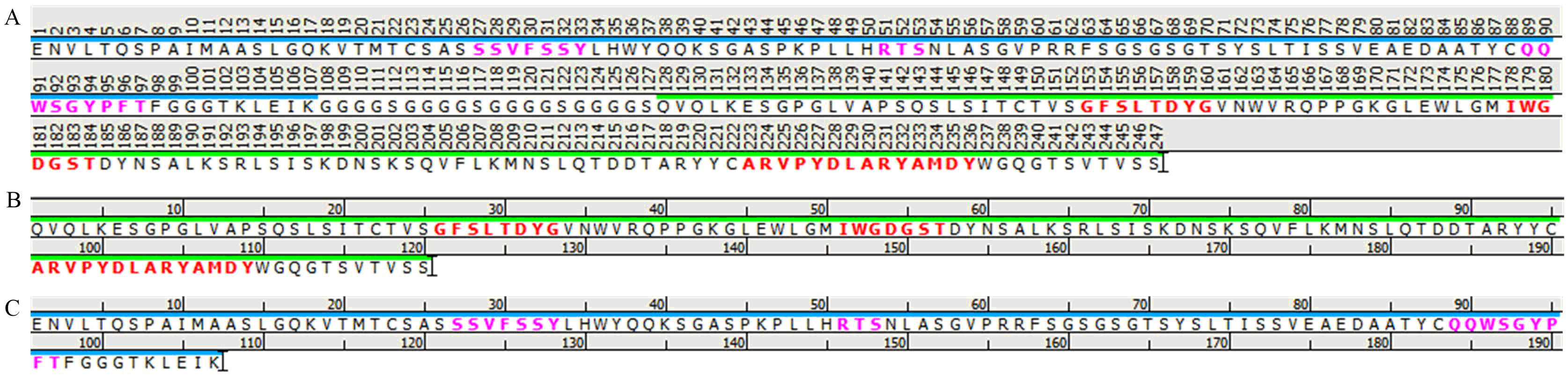


**Supplementary Figure 6.** Evaluation of scFv spatial structure model. (A) Ramachandran plot of scfv model. (Blue represents the optimal area and purple represents the allowable area). (B) Profile-3D results of scfv model.
